# Reducing medication burden and improving pressure injury outcomes in older emergency patients: a pharmacist-nurse collaboration

**DOI:** 10.3389/fmed.2026.1863115

**Published:** 2026-06-23

**Authors:** Lianhua Ji, Li Li, Juan Zhang, Sujuan Zhang, Zhonghua Fu

**Affiliations:** 1 Department of Emergency, Henan Provincial People's Hospital, People's Hospital of Zhengzhou University, Henan University, Zhengzhou, Henan, China; 2 Clinical Pharmacy, Henan Provincial People's Hospital, People's Hospital of Zhengzhou University, Henan University, Zhengzhou, Henan, China

**Keywords:** Drug Burden Index, emergency department, Medication Regimen Complexity Index, pharmacist-nurse collaboration, pressure injury, stratified nursing care, older adults

## Abstract

**Background:**

Older adults admitted for acute conditions with pre-existing pressure injuries (PIs) often experience poor prognoses and are frequently affected by multimorbidity and polypharmacy. Medication burden and complex treatment regimens may further impede recovery. However, pharmacist-led comprehensive medication management integrated with stratified nursing care for PI management in emergency wards remains insufficiently investigated.

**Methods:**

This retrospective before–after cohort study was conducted in a tertiary hospital. Older patients with pre-existing PIs at admission were allocated to a pre-implementation cohort (2024, *n* = 165) and a post-implementation cohort (2025, *n* = 175). The intervention consisted of a pharmacist-involved multidisciplinary stratified care model, including medication reconciliation, regimen optimization, risk-stratified nursing care, repositioning protocols, pressure off-loading measures, nutritional support, and health education. Outcomes were evaluated using changes in Braden scores, wound healing status, clinical recovery, length of stay, Charlson Comorbidity Index (CCI), Drug Burden Index (DBI), Medication Regimen Complexity Index (MRCI), number of medications, and medication cost proportion. A contemporaneous non-PI cohort (200 vs. 200) was randomly selected as a reference group to exclude systemic temporal trends.

**Results:**

Compared with the reference group, patients with PIs exhibited higher CCI, DBI, and MRCI scores and poorer clinical outcomes. Baseline characteristics were comparable between groups. Following implementation, the proportion of medication costs and daily MRCI scores decreased significantly (both *P* < 0.05). The mean number of prescribed agents decreased by 3.37 and DBI declined by 25.30%, though neither reached statistical significance. The post-implementation cohort demonstrated higher rates of clinical recovery (10.3 vs. 1.2%, *P* = 0.002), PI improvement (87.4 vs. 77.0%, *P* = 0.041), and Braden score improvement (78.9 vs. 53.9%, *P* < 0.001). Length of stay decreased by 1.1 days (*P* = 0.142). No significant changes were observed in the non-PI reference cohort over the corresponding timeframe.

**Conclusions:**

A pharmacist-involved, multidisciplinary stratified care model effectively reduced medication burden and regimen complexity while improving wound healing and functional outcomes in older emergency patients with PIs, providing a feasible and pragmatic strategy for optimizing PI management.

## 1 Introduction

Pressure injuries (PIs) remain a prevalent and clinically significant complication among older adults, contributing to prolonged hospitalization, functional decline, increased healthcare expenditures, and elevated mortality (1, 2). Vulnerability is especially pronounced in emergency department (ED) patients, in whom advanced age, multimorbidity, immobility, and acute physiological instability converge to precipitate rapid tissue breakdown (3). Despite the extensive implementation of clinical strategies aimed at enhancing treatment outcomes—such as risk assessment scales, wound assessment and staging tools, pressure-relieving devices, and wound dressings—the prevention and management of community-acquired PIs in older adults continue to be suboptimal (4, 5).

Polypharmacy further compounds this risk (6, 7). Anticholinergic and sedative agents impair cognition, mobility, and tissue perfusion, thereby delaying wound healing, whereas complex regimens increase treatment burden, diminish adherence, and reduce care efficiency (8). Although indices such as the Charlson Comorbidity Index (CCI), Drug Burden Index (DBI) (9), and Medication Regimen Complexity Index (MRCI) (10) enable objective quantification of clinical and pharmacologic burden (11, 12), these metrics are seldom integrated into routine PI management in ED settings.

Multidisciplinary models incorporating nursing, pharmacy, and nutritional support have demonstrated benefit in frail populations (13, 14). However, evidence for pharmacist-integrated, stratified care pathways targeting ED patients with established PIs remains limited, and whether reducing medication burden translates into improved wound and functional outcomes remains unclear. Accordingly, we evaluated a pharmacist-integrated, risk-stratified care model for older ED patients with PIs, hypothesizing that this approach would reduce medication burden and regimen complexity while improving wound healing and hospitalization outcomes.

## 2 Methods

### 2.1 Data source and study design

This retrospective before–after cohort study was conducted in the emergency department of a tertiary teaching hospital. Research data were obtained through a formal request and extracted from the hospital's Meikang Clinical Intelligence System after de-identification. The dataset covered electronic medical records of patients admitted between January 1, 2024, and December 31, 2025. The study ensured that personal identifying information of the sample was not accessible to the researchers.

Older adults presenting with pre-existing PIs were identified and stratified by calendar year into a pre-implementation cohort (2024) and a post-implementation cohort (2025). To provide a contemporaneous reference group, 400 admissions without PIs (200 per year) were randomly sampled from the same ward.

Extracted variables comprised demographic characteristics (sex, age, body mass index, insurance status), clinical indicators (temperature, comorbidities, diagnoses, and interdepartmental transfers), hospitalization outcomes (length of stay, wound status, and changes in Braden scores), and medication-related parameters (drug classes, number of medications, and prescribing patterns).

### 2.2 Eligibility criteria

Patients were included if they were admitted to the emergency ward, received PI–specific treatment within 24 h of admission, had documented PI nursing charges, and were aged ≥ 60 years. Exclusion criteria were hospital-acquired PIs, fewer than three documented PI nursing interventions, and incomplete Braden or nursing records. Clinical outcomes were determined according to the attending physician's discharge assessment.

### 2.3 Intervention

Starting January 2025, a pharmacist-led multidisciplinary care model was implemented. Clinical pharmacists participated in routine ward rounds, medication counseling, and structured medication review. In collaboration with nursing staff, patients with community-acquired PIs underwent structured follow-up and regimen optimization.

Level of care was stratified according to Braden risk scores and wound stage. Patients at moderate-to-high risk or with stage I injuries were managed by designated nurses, whereas those with stage II or higher injuries, or with persistently high risk, were referred for multidisciplinary team (MDT) consultation. The MDT included clinical pharmacists (general ward-based and nutrition-specialized), wound therapists, PI nurses, and rehabilitation specialists, implementing individualized and dynamically adjusted care plans.

Core components included: 1) Regular pharmacist-led medication reconciliation and regimen optimization with physician feedback; 2) Shift-based full-body skin inspections supported by electronic high-risk alerts; 3) Individualized repositioning protocols (30-min alternating lateral positioning; head-of-bed elevation ≤ 30 °); 4) Standardized pressure redistribution using air mattresses, positioning devices, and protective dressings; 5) Structured incontinence management and skin protection; 6) Nutritional screening with tailored enteral or parenteral support when indicated; 7) Patient and caregiver education combined with psychosocial counseling.

### 2.4 Measures

#### 2.4.1 Pressure injury risk and staging

PI risk was assessed using the Braden Scale (15), a validated six-domain scale (sensory perception, moisture, activity, mobility, nutrition, and friction/shear) with scores ranging from six to 23; lower scores indicate greater risk. To minimize interdepartmental variability, the mean value of recorded scores was used. PI wounds were staged according to the 2019 NPIA/EPUAP classification (stages I–IV, unstageable, or deep tissue injury) (16). Staging and outcomes were independently evaluated by two senior nurses, with discrepancies resolved by a head nurse.

#### 2.4.2 Comorbidity burden

Comorbidity was quantified using the Charlson Comorbidity Index (CCI) (17). Diagnoses were mapped to 19 predefined categories using the ICD-10 coding algorithm proposed by Quan et al. (18), with age-adjusted weighting. Conditions not included in the CCI were entered separately as covariates. Higher scores indicate greater disease burden.

#### 2.4.3 Drug burden

Cumulative pharmacological burden was quantified using the Drug Burden Index (DBI) (19), a dose-standardized measure of anticholinergic and sedative medication exposure. DBI = Σ [D / (δ + D)], where D represents the daily dose and δ the minimum recommended effective dose. Anticholinergic and sedative agents were identified using established classifications, and individual drug contributions were summed to obtain total DBI. Medications administered solely for diagnostic or procedural purposes were not considered.

Regimen complexity was evaluated using the validated Medication Regimen Complexity Index (MRCI) (20), comprising 65 weighted items across dosage form (32 items), dosing frequency (23 items), and additional instructions (10 items). Scores for each medication were summed to derive total regimen complexity, with higher scores indicating greater complexity. Nonstandard administration routes were scored by consensus among pharmacists and nurses based on operational burden. Periods of prolonged sedation for immobilization were excluded. To account for variations in length of stay, a daily MRCI was calculated as the total MRCI score divided by hospitalization days.

### 2.5 Statistical analysis

All analyses were performed with SPSS version 27.0 (IBM Corp., Armonk, NY, USA). Normally distributed continuous variables are presented as mean ± standard deviation and were compared using independent-sample *t*-tests. Categorical variables are summarized as frequencies and percentages and were compared using chi-square tests. To control for potential confounders, multivariable linear regression was used for continuous outcomes (daily MRCI and medication cost ratio), multivariable ordinal logistic regression for ordered categorical outcomes (PI outcome), and multivariable multinomial logistic regression for multinomial outcomes (disease outcome and Braden score change), with implementation period (2025 vs. 2024) as the primary independent variable, adjusting for age, sex, BMI, body temperature, hypertension, diabetes, hyperlipidemia, risk level, wound stage, and CCI score. A two-sided *P* value < 0.05 was considered statistically significant.

## 3 Results

### 3.1 Baseline characteristics

A total of 340 eligible older adults with pre-existing PIs were included in the final analysis, comprising 165 patients in the pre-implementation cohort and 175 in the post-implementation cohort (Table 1). Baseline demographic and clinical characteristics were comparable between groups, indicating baseline equivalence prior to intervention exposure.

**Table 1:** Baseline characteristics of participants.

Variable	Category	Pre-implementation (*n* = 165)	Post-implementation (*n* = 175)	Effect Size (95%CI)	*P* value
Sex	Male	97 (58.8%)	86 (49.1%)	0.10 (−0.01, 0.20)	0.075
	Female	68 (41.2%)	89 (50.9%)		
Age (years)	60–74	81 (49.1%)	78 (44.3%)	−0.08 (−0.29, 0.14)	0.485
	75–89	78 (47.3%)	84 (47.7%)		
	≥90	6 (3.6%)	13 (7.4%)		
	mean ± SD	75.32 ± 8.50	75.96 ± 8.35		
BMI (kg/m^2^)	< 18.5	20 (12.1%)	24 (13.7%)	0.04 (−0.17, 0.26)	0.660
	18.5–23.9	94 (57.0%)	99 (56.6%)		
	≥24.0	51 (30.9%)	52 (29.7%)		
	Mean ± SD	22.10 ± 5.68	21.83 ± 5.79		
Insurance type	Resident insurance	105 (63.6%)	120 (68.6%)	0.08 (−0.02, 0.19)	0.307
	Employee insurance	52 (31.5%)	43 (24.6%)		
	Self-pay	8 (4.9%)	12 (6.9%)		
Department transfer	Yes	21 (12.7%)	33 (18.9%)	0.08 (−0.02, 0.19)	0.122
Body temperature	< 37.3 °C	88 (53.3%)	95 (54.3%)	0.11 (0.00, 0.21)	0.138
	37.3–38.4 °C	35 (21.2%)	49 (28.0%)		
	≥38.5 °C	42 (25.5%)	31 (17.7%)		
Medical history	Hypertension	98 (59.4%)	95 (54.3%)	−0.05 (−0.16, 0.06)	0.342
	Diabetes	72 (43.6%)	84 (48.0%)	0.04 (−0.06, 0.15)	0.420
	Hyperlipidemia	51 (30.9%)	47 (26.9%)	−0.05 (−0.15, 0.06)	0.410
	Allergy	28 (17.0%)	19 (10.9%)	−0.09 (−0.19, 0.02)	0.103
CCI score	(mean ± SD)	3.45 ± 1.82	3.67 ± 1.89	−0.12 (−0.33, 0.10)	0.289
Risk level	Low	21 (12.7%)	29 (16.6%)	0.12 (0.01, 0.22)	0.196
	Moderate	69 (41.8%)	57 (32.6%)		
	High	74 (44.9%)	85 (48.6%)		
	Very high	1 (0.6%)	4 (2.3%)		
Wound stage	Stage I	138 (83.6%)	148 (84.6%)	0.02 (−0.08, 0.13)	0.981
	Stage II	18 (10.9%)	17 (9.7%)		
	Stage III	4 (2.4%)	4 (2.3%)		
	Stage IV^*^	5 (3.0%)	6 (3.4%)		

BMI, body mass index; CCI, Charlson Comorbidity Index.

Data are presented as *n* (%) or mean ± standard deviation (SD). *P* values were calculated using the chi-square test or independent-samples *t*-test.

Stage IV includes Stage IV, unstageable pressure injury, and deep tissue pressure injury.

No significant between-group differences were observed in sex distribution (male: 58.8 vs. 49.1%, *P* = 0.075), mean age (75.32 ± 8.50 vs. 75.96 ± 8.35 years, *P* = 0.485), or BMI (22.10 ± 5.68 vs. 21.83 ± 5.79 kg/m^2^, *P* = 0.660). Likewise, insurance type, interdepartmental transfer rate, presence of fever at admission, and prior medical history—including hypertension, diabetes mellitus, hyperlipidemia, and allergy history—showed no significant differences. Comorbidity burden, quantified by the CCI, was comparable (3.45 ± 1.82 vs. 3.67 ± 1.89, *P* = 0.289), as were distributions of PI risk classification and wound stage. Overall, no significant differences were detected across baseline variables (all *P* > 0.05), supporting the appropriateness of subsequent outcome comparisons.

### 3.2 Medication utilization metrics

Medication-related parameters showed significant improvements following implementation of the pharmacist-involved multidisciplinary intervention. The proportion of medication expenditures relative to total hospitalization costs decreased significantly in the post-implementation cohort compared with the pre-implementation cohort (21.10 ± 10.13% vs. 23.93 ± 11.17%, *P* = 0.015). In parallel, treatment regimen complexity, assessed using the daily MRCI, demonstrated a significant reduction (41.05 ± 19.03 vs. 46.23 ± 20.19, *P* = 0.015), indicating simplified prescribing structures and administration schedules.

Additional reductions were observed in overall medication burden. The mean number of prescribed agents decreased by 3.37 medications (37.27 ± 19.11 vs. 40.64 ± 19.31), and DBI scores declined by 25.30% (2.48 ± 3.20 vs. 3.32 ± 5.06). Although these changes appeared clinically meaningful, statistical significance was not achieved (Table 2).

**Table 2:** Medication burden and regimen complexity between cohorts.

Variable	Category	Pre-implementation (*n* = 165)	Post-implementation (*n* = 175)	Effect size (95%CI)	*P* value
Number of medications	Mean ± SD	40.64 ± 19.31	37.27 ± 19.11	0.18 (−0.04, 0.39)	0.107
Medication cost ratio, (%)	Mean ± SD	23.93 ± 11.17	21.10 ± 10.13	0.27 (0.05, 0.48)	0.015
Daily MRCI	Mean ± SD	46.23 ± 20.19	41.05 ± 19.03	0.26 (0.05, 0.48)	0.015
DBI category	0	86 (52.1%)	102 (58.3%)	0.17 (−0.05, 0.38)	0.224
	0– < 2	44 (26.7%)	45 (25.7%)		
	≥2	35 (21.2%)	28 (16.0%)		
	Mean ± SD	3.32 ± 5.06	2.48 ± 3.20		

Daily MRCI, mean daily Medication Regimen Complexity Index (total MRCI divided by length of stay); DBI, Drug Burden Index; DBI category, stratified as 0 (no exposure), 0– < 2 (moderate burden), and ≥2 (high burden).

### 3.3 Changes in braden scores

**Figure 1:**
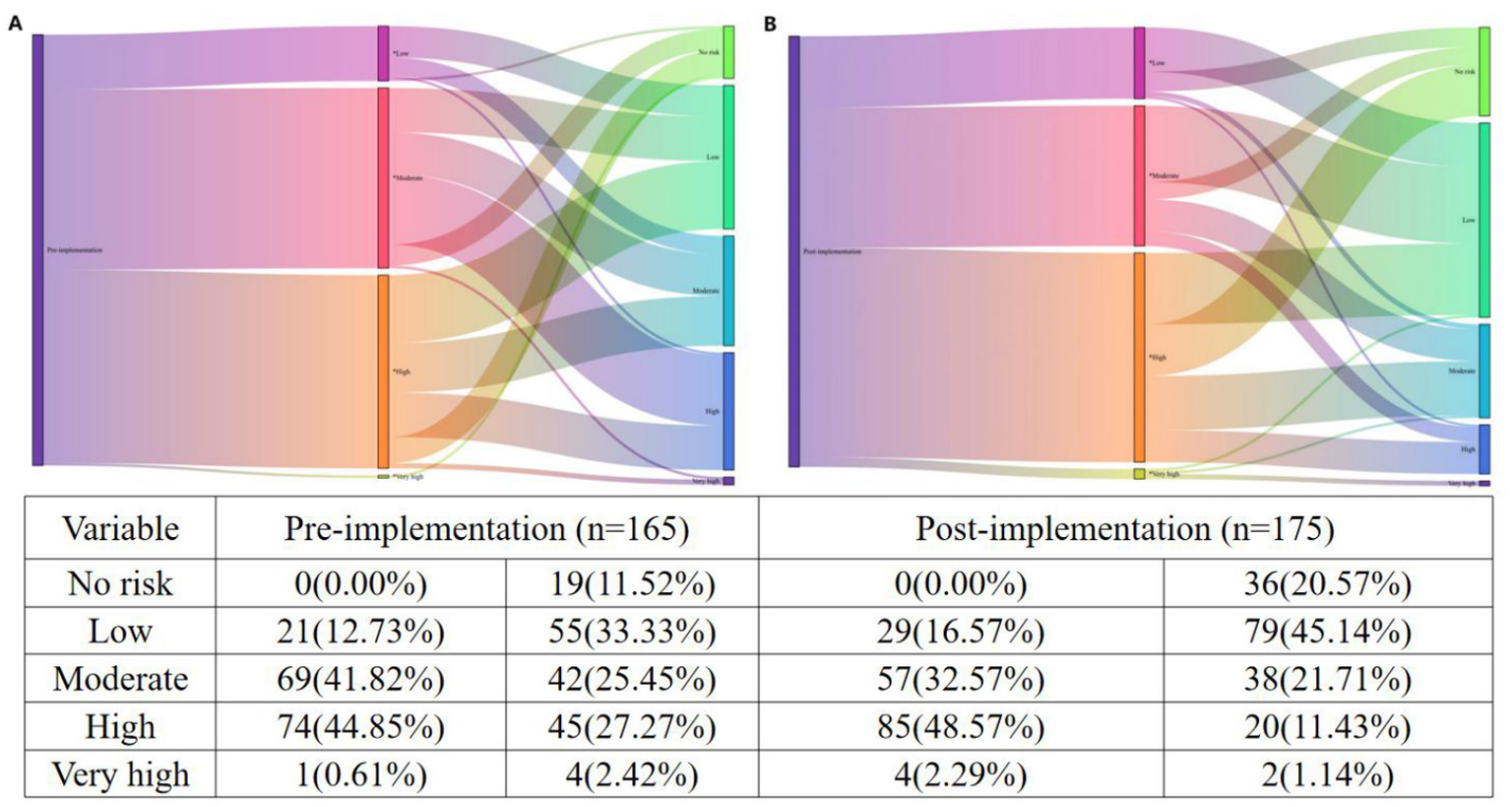
Transitions of Braden risk levels from admission to discharge in the pre- and post-implementation cohorts. Sankey diagrams show the distribution and transitions of pressure injury risk levels from admission to discharge in the pre-implementation **(B)** and post-implementation **(A)** cohorts. The width of each flow is proportional to the number of patients moving between categories. Risk levels were classified according to the Braden Scale as no risk, low, moderate, high, and very high.

Risk stratification based on the Braden Scale assessment showed a shift toward lower PI risk categories following intervention. At admission, the proportions of patients classified as moderate risk (41.82 vs. 32.57%) and high risk (44.85 vs. 48.57%) were comparable between cohorts. After the intervention period, a redistribution toward more favorable risk levels was observed. The proportions classified as no risk increased from 12.12% to 20.57%, and those categorized as low risk increased from 33.33 to 45.14%, whereas the proportion of patients classified as high risk decreased from 27.27 to 11.43%. Overall, transitions from moderate-to-high risk to no or low risk occurred more frequently in the post-implementation cohort, suggesting improved skin integrity and reduced vulnerability to PI progression (Figure 1).

### 3.4 Clinical and wound outcomes

Implementation of the integrated care model was accompanied by improvements in both clinical recovery and wound-related outcomes. The overall disease recovery rate increased (10.3 vs. 1.2%, *P* = 0.002). Similarly, PI improvement was more frequent in the post-implementation cohort (87.4 vs. 77.0%, *P* = 0.041), and Braden score improvement occurred more often after intervention (78.9 vs. 53.9%, *P* < 0.001). Length of stay decreased by 1.1 days on average (11.91 ± 7.61 vs. 13.21 ± 8.64 days), suggesting improved inpatient efficiency. The incidence of clinical deterioration or death decreased slightly (10.9 vs. 12.1%) but did not achieve statistical significance (Table 3).

**Table 3:** Clinical outcomes of patients before and after implementation.

Variable	Category	Pre-implementation (*n* = 165)	Post-implementation (*n* = 175)	Effect size (95%CI)	*P* value
Length of stay (days)	mean ± SD	13.21 ± 8.64	11.91 ± 7.61	0.16 (−0.05, 0.37)	0.142
Disease outcome	Recovered	2 (1.2%)	18 (10.3%)	0.19 (0.09, 0.29)	0.002
	Improved	143 (86.7%)	138 (78.9%)		
	Deteriorated or died	20 (12.1%)	19 (10.9%)		
PI outcome	Improved	127 (77.0%)	153 (87.4%)	0.14 (0.03, 0.24)	0.041
	Unchanged	28 (17.0%)	16 (9.1%)		
	Deteriorated	10 (6.1%)	6 (3.4%)		
ΔBraden score	Increased	89 (53.9%)	138 (78.9%)	0.29 (0.19, 0.39)	< 0.001
	No change	27 (16.4%)	22 (12.6%)		
	Decreased	49 (29.7%)	15 (8.6%)		

PI, pressure injury; PI outcome, clinical outcome of pressure injury (improved, stable, or deteriorated); Δ Braden score, change in Braden Scale score during hospitalization (increase, decrease, or unchanged).

### 3.5 Multivariable analysis

To further evaluate the independent effect of the intervention, multivariable regression analyses were performed adjusting for baseline demographic and clinical characteristics (Supplementary Tables 1–5). After controlling for age, sex, BMI, temperature, comorbidities, risk level, wound stage, and CCI score, the post-implementation period remained independently associated with lower daily MRCI (B = −5.77, 95% CI −9.83 to −1.71, *P* = 0.006; Supplementary Table 1) and lower medication cost ratio (B = −0.03, 95% CI −0.05 to −0.01, *P* = 0.003; Supplementary Table 2). For clinical outcomes, the intervention was associated with significantly higher odds of PI improvement (adjusted OR 2.20, 95% CI 1.20–4.01, *P* = 0.010; Supplementary Table 3) and higher odds of Braden score increase vs. decrease (adjusted OR 6.67, 95% CI 3.45–12.86, *P* < 0.001; Supplementary Table 5). The odds of clinical recovery were also significantly higher in the post-implementation cohort (Supplementary Table 4).

### 3.6 Negative control (reference cohort)

To evaluate potential secular trends, a contemporaneous reference cohort of 400 non-PI patients (200 per period) was analyzed. No significant differences were observed between the pre- and post-implementation groups across any measured variables. CCI scores, number of medications, medication cost proportion, length of stay, DBI score, and daily MRCI showed no significant changes. Clinical outcomes likewise did not differ (*P* = 0.86; Table 4). These findings suggest that the improvements observed among PI patients were unlikely to reflect secular trends or system-wide changes unrelated to the intervention.

**Table 4:** Baseline characteristics of the concurrent reference cohort.

Variable	Category	Pre-implementation (*n* = 200)	Post-implementation (*n* = 200)	Effect Size (95%CI)	*P* value
CCI score	(mean ± SD)	0.55 ± 0.87	0.60 ± 0.94	0.06 (−0.25, 0.14)	0.581
Number of medications	Mean ± SD	17.39 ± 8.18	17.30 ± 7.32	0.01 (−0.18, 0.21)	0.903
Medication cost ratio (%)	Mean ± SD	15.67 ± 9.68	15.53 ± 8.81	0.01 (−0.18, 0.21)	0.888
Length of stay (days)	mean ± SD	5.78 ± 2.60	5.79 ± 2.46	−0.00 (−0.20, 0.19)	0.968
Disease outcome	Recovered	2 (1.0%)	3 (1.5%)	0.03 (−0.08, 0.13)	0.86
	Improved	193 (96.5%)	191 (95.5%)		
	Deteriorated or died	5 (2.5%)	6 (3.0%)		
DBI category	0	164 (82.0%)	171 (85.5%)	0.06 (−0.13, 0.26)	0.522
	0– < 2	36 (18.0%)	29 (14.5%)		
	Mean ± SD	0.15 ± 0.38	0.13 ± 0.35		
Daily MRCI	Mean ± SD	27.82 ± 13.32	27.96 ± 18.96	−0.01 (−0.21, 0.19)	0.932

## 4 Discussion

In this real-world cohort study of older emergency department patients with pre-existing PIs, implementation of a pharmacist-integrated, risk-stratified multidisciplinary care pathway was associated with consistent and clinically meaningful improvements across medication-related, functional, and wound outcomes. Compared with the pre-implementation cohort, the post-implementation group demonstrated significant reductions in the proportion of medication expenditures (23.93 vs. 21.10%), daily MRCI (46.23 vs. 41.05), number of prescribed agents (−3.37 medications on average), and DBI (−25.3%), accompanied by higher rates of Braden score improvement (78.9 vs. 53.9%), greater wound recovery (87.4 vs. 77.0%), and a shorter length of stay (−1.1 days). Notably, these alterations were absent in the non-PI control cohort throughout the corresponding timeframe. The incorporation of an external control group effectively mitigated the potential impact of temporal bias and extraneous variables, thereby substantiating that the advantages observed in this study are predominantly attributable to the collaborative intervention between pharmacists and nurses. Furthermore, multivariable analyses adjusting for baseline demographic and clinical characteristics confirmed that the post-implementation period remained independently associated with improved PI outcomes, enhanced Braden scores, reduced MRCI, and lower medication cost ratios (Supplementary Tables 1–5), strengthening the argument that these benefits are attributable to the collaborative intervention rather than confounding variables.

Polypharmacy, particularly the use of anticholinergic drugs and sedatives, has been associated with delirium, cognitive impairment, falls, and reduced mobility (21, 22). The Drug Burden Index (DBI) quantifies the cumulative impact of these medications on function (23, 24), and medication-related dysfunction reduces patients' ability to change position independently and exacerbates microvascular ischemia. Previous studies have shown (25–27) that clinical pharmacist intervention can reduce inappropriate prescribing and adverse drug events, thereby lowering the risk of polypharmacy in older patients. The complexity of medication regimens further contributes to reduced patient adherence (28, 29), and simplifying medication regimens can improve patient adherence and treatment continuity. Medication optimization is a key determinant of functional frailty in older adults. In this study, the concurrent reduction in DBI and MRCI was accompanied by improvements in Braden scores and wound healing outcomes, suggesting that pharmacist-led interventions can both enhance physiological reserve and functional capacity and indirectly improve tissue perfusion, thereby promoting patient outcomes.

By providing nutritional supplementation (particularly adequate protein and caloric intake) to PI patients with nutritional deficiencies, pharmacists can accelerate wound healing and reduce the severity of PI (30). Pharmacist interventions reduce the use of anticholinergic and sedative medications, lower the risk of falls, and promote early mobility and rehabilitation exercises, thereby improving blood circulation and tissue oxygen delivery, which facilitates early recovery. Pharmacist interventions can reduce the number and frequency of medications, thereby optimizing nursing work and freeing up nursing resources for the care of PI patients (31, 32). Through a tiered model, providing personalized care to PI patients enables the precise allocation of nursing resources. Enhanced nursing intensity and risk stratification form a complementary mechanism. The pharmacist-nurse collaboration model also promotes and strengthens multidisciplinary cooperation. Pharmacists optimize medication regimens, nurses implement continuous preventive care, physicians manage comorbidities, and rehabilitation specialists facilitate systemic recovery. Previous studies have also confirmed that a coordinated multidisciplinary team approach for elderly patients with complex conditions, multiple comorbidities, and polypharmacy is superior to isolated, single-intervention measures (33).

Several limitations must be acknowledged in this study. Firstly, the retrospective design limits the ability to draw definitive causal inferences. Although the contemporaneous non-PI reference cohort helps mitigate temporal bias arising from policy changes, staffing adjustments, and system-wide improvements, the possibility of residual confounding cannot be entirely dismissed. Secondly, the study's single-center setting at a tertiary hospital may restrict the generalizability of the findings to other institutions, particularly those with different staffing structures or limited pharmacy and wound care resources. Thirdly, it was not possible to disentangle the contributions of individual intervention components, such as pharmacist optimization, nursing intensification, risk stratification, and multidisciplinary team coordination. Therefore, prospective, multicenter trials incorporating process evaluation designs are necessary to elucidate the relative effects of these components.

## 5 Conclusion

In conclusion, a pharmacist-integrated, risk-stratified multidisciplinary model was feasible in the emergency setting and was associated with reduced medication burden, simplified regimens, improved functional status, enhanced wound recovery, and shorter hospitalization among older adults with PIs. These findings support a multidimensional framework in which pharmacologic optimization, nursing intensification, risk stratification, and team-based coordination interact synergistically to improve outcomes. Future prospective studies should validate these mechanisms and determine the relative contribution of each component to maximize effectiveness and scalability.

## Data Availability

The raw data supporting the conclusions of this article will be made available by the authors, without undue reservation.
