# Supplementary material for: Reducing medication burden and improving pressure injury outcomes in older emergency patients: a pharmacist-nurse collaboration
Source: Front Med (Lausanne). 2026 Jun 23;13:1863115. doi: 10.3389/fmed.2026.1863115 (PMC13337471; doi:10.3389/fmed.2026.1863115)
Supplement: Supplementary file 1 [file Table_1.docx]

**Table S1:**Multivariable Linear Regression Analysis of Factors Associated with Mean MRCI

| **Variable** | **B** | **SE** | **β** | **t** | **P value** |
| --- | --- | --- | --- | --- | --- |
| Constant | 63.63 | 12.05 | 0 | 5.28 | **＜0.001** |
| Sex | 0.17 | 2.16 | 0 | 0.08 | 0.937 |
| Age | -0.17 | 0.15 | -0.07 | -1.18 | 0.241 |
| BMI | -0.17 | 0.18 | -0.05 | -0.95 | 0.342 |
| Temperature | 4.71 | 2.16 | 0.12 | 2.18 | **0.03** |
| Hypertension | 0.66 | 2.15 | 0.02 | 0.31 | 0.758 |
| Diabetes | 5.86 | 2.2 | 0.15 | 2.67 | **0.008** |
| Hyperlipidemia | -7.59 | 2.47 | -0.17 | -3.08 | **0.002** |
| Risk level | -6.65 | 2.98 | -0.12 | -2.23 | **0.026** |
| Wound stage | 2.7 | 3.46 | 0.04 | 0.78 | 0.437 |
| CCI score | -0.17 | 0.68 | -0.02 | -0.25 | 0.805 |
| Group | -5.77 | 2.07 | -0.15 | -2.79 | **0.006** |

B, unstandardized regression coefficient; SE, standard error; β, standardized regression coefficient; t, t-statistic.

**Table S2:**Multivariable Linear Regression Analysis of Factors Associated with Medication cost ratio

| **Variable** | **B** | **SE** | **β** | **t** | **P value** |
| --- | --- | --- | --- | --- | --- |
| Constant | 0.23 | 0.07 | 0 | 3.57 | **＜0.001** |
| Sex | 0.00 | 0.01 | -0.02 | -0.28 | 0.781 |
| Age | 0.00 | 0.00 | 0.03 | 0.45 | 0.657 |
| BMI | 0.00 | 0.00 | -0.03 | -0.53 | 0.595 |
| Temperature | 0.02 | 0.01 | 0.11 | 1.99 | **0.048** |
| Hypertension | 0.01 | 0.01 | 0.04 | 0.75 | 0.456 |
| Diabetes | 0.00 | 0.01 | 0.02 | 0.31 | 0.753 |
| Hyperlipidemia | -0.07 | 0.01 | -0.31 | -5.54 | **＜0.001** |
| Risk level | -0.01 | 0.02 | -0.02 | -0.39 | 0.698 |
| Wound stage | -0.01 | 0.02 | -0.04 | -0.68 | **0.50** |
| CCI score | 0.01 | 0.00 | 0.09 | 1.47 | 0.143 |
| Group | -0.03 | 0.01 | -0.15 | -2.96 | **0.003** |

B, unstandardized regression coefficient; SE, standard error; β, standardized regression coefficient; t, t-statistic.

**Table S**3: Ordered logistic regression analysis of the PI Outcome

| Variable | β | SE | OR (95% CI) | P-value |
| --- | --- | --- | --- | --- |
| Sex(Female vs Male) | 0.05 | 0.31 | 1.05(0.57–1.94) | 0.866 |
| Age (per 1 year) | 0.06 | 0.02 | 1.06 (1.02–1.11) | **0.005** |
| BMI | 0 | 0.03 | 1.00 (0.95–1.05) | 0.988 |
| CCI index | 0.09 | 0.1 | 1.09 (0.89–1.34) | 0.398 |
| ****Group (2025 vs 2024)**** | 0.79 | 0.31 | 2.20 (1.20–4.01) | **0.01** |
| Temperature(Normal vs Fever) | -0.08 | 0.31 | 0.92 (0.50–1.70) | 0.797 |
| Hypertension (N vs Y) | 0.08 | 0.31 | 1.08 (0.59–2.00) | 0.796 |
| Diabetes (N vs Y) | 0.06 | 0.32 | 1.06 (0.56–1.98) | 0.863 |
| Hyperlipidemia (N vs Y) | 0.28 | 0.37 | 1.32 (0.64–2.75) | 0.451 |
| Risk level(low vs low+) | -0.93 | 0.56 | 0.40（0.13–1.19) | 0.098 |
| Wound stage(Ӏ vs Ӏ+) | -0.72 | 0.43 | 0.49(0.21–1.12) | 0.091 |

CI：Confidence interval; OR：Odds ratio; SE：Standard error; CCI：Charlson Comorbidity Index; N/Y：No/Yes;I vs I+：Wound stage I vs higher stages;low vs low+：Low risk vs higher risk.

Table S4:Multinomial Logistic Regression Analysis of Factors Associated with Clinical Outcomes

| Variable | Improvement vs. Deteriorated or died | | Cure vs. Deteriorated or died | |
| --- | --- | --- | --- | --- |
|  | OR (95% CI) | P-value | OR (95% CI) | P-value |
| Age (per 1 year) | 0.99 (0.94–1.04) | 0.729 | 1.04 (0.95–1.14) | 0.370 |
| BMI | 1.01 (0.94–1.07) | 0.863 | 0.97 (0.88–1.08) | 0.619 |
| CCI index | 0.85 (0.67–1.09) | 0.205 | 0.52 (0.35–0.78) | **0.002** |
| Group (2025 vs 2024) | 0.86 (0.42–1.78) | 0.690 | 0.07 (0.01–0.39) | **0.002** |
| Sex(Female vs Male) | 0.73 (0.35–1.54) | 0.413 | 0.26 (0.07–0.98) | **0.046** |
| Temperature(Normal vs Fever) | 1.17 (0.55–2.46) | 0.689 | 0.22 (0.05–0.89) | **0.034** |
| Hypertension (N vs Y) | 1.08 (0.51–2.30) | 0.833 | 1.03 (0.29–3.65) | 0.961 |
| Diabetes (N vs Y) | 0.95 (0.44–2.08) | 0.901 | 0.70 (0.20–2.49) | 0.584 |
| Hyperlipidemia (N vs Y) | 0.21 (0.07–0.63) | **0.005** | 0.10 (0.02–0.55) | **0.008** |
| Risk level (low vs low+) | 0.15 (0.07–0.35) | **＜0.001** | 0.46 (0.11–1.88) | 0.281 |
| Wound stage (I vs I+) | 1.24 (0.42–3.71) | 0.700 | 0.95 (0.17–5.17) | 0.949 |

CI：Confidence interval; OR：Odds ratio; SE：Standard error; CCI：Charlson Comorbidity Index; N/Y：No/Yes;I vs I+：Wound stage I vs higher stages;low vs low+：Low risk vs higher risk; OR＞1 indicates a higher probability of the respective outcome compared with "Deteriorated or died";.

**T**able S5: Multinomial Logistic Regression Analysis of Factors Associated with Braden score

| **Variable** | **Stable vs. Decreased** | | **Increased vs. Decreased** | |
| --- | --- | --- | --- | --- |
|  | OR (95% CI) | P-value | OR (95% CI) | P-value |
| Age (per 1 year) | 1.06 (1.00–1.12) | 0.066 | 1.02 (0.98–1.07) | 0.325 |
| BMI | 1.01 (0.95–1.08) | 0.676 | 1.03 (0.98–1.08) | 0.302 |
| CCI index | 0.93 (0.71–1.22) | 0.595 | 0.86 (0.70–1.06) | 0.156 |
| Group (2025 vs 2024) | 0.36 (0.16–0.85) | **0.019** | 0.15 (0.07–0.29) | **＜0.001** |
| Sex(Female vs Male) | 0.62 (0.28–1.41) | 0.257 | 0.54 (0.29–1.04) | 0.063 |
| Temperature(Normal vs Fever) | 0.43 (0.19–0.97) | **0.043** | 0.78 (0.40–1.49) | 0.444 |
| Hypertension (N vs Y) | 0.88 (0.39–1.99) | 0.752 | 0.82 (0.44–1.56) | 0.551 |
| Diabetes (N vs Y) | 0.69 (0.29–1.60) | 0.381 | 0.85 (0.44–1.64) | 0.627 |
| Hyperlipidemia (N vs Y) | 1.39 (0.55–3.53) | 0.493 | 1.19 (0.59–2.39) | 0.628 |
| Risk level (low vs low+) | 1.36 (0.55–3.40) | 0.505 | 0.22 (0.09–0.50) | **＜0.001** |
| Wound stage (I vs I+) | 0.92 (0.26–3.27) | 0.895 | 0.86 (0.30–2.46) | 0.771 |

CI：Confidence interval; OR：Odds ratio; SE：Standard error; CCI：Charlson Comorbidity Index; N/Y：No/Yes;I vs I+：Wound stage I vs higher stages;low vs low+：Low risk vs higher risk; OR＞1 indicates a higher probability of the respective outcome compared with "Decreased".
